# Supplementary material for: Modulation of sensory prediction error in Purkinje cells during visual feedback manipulations
Source: Nat Commun. 2018 Mar 15;9:1099. doi: 10.1038/s41467-018-03541-0 (PMC5854574; doi:10.1038/s41467-018-03541-0)
Supplement: Supplementary file 1 — Supplementary Information(PDF 288 kb) [file 41467_2018_3541_MOESM1_ESM.pdf]

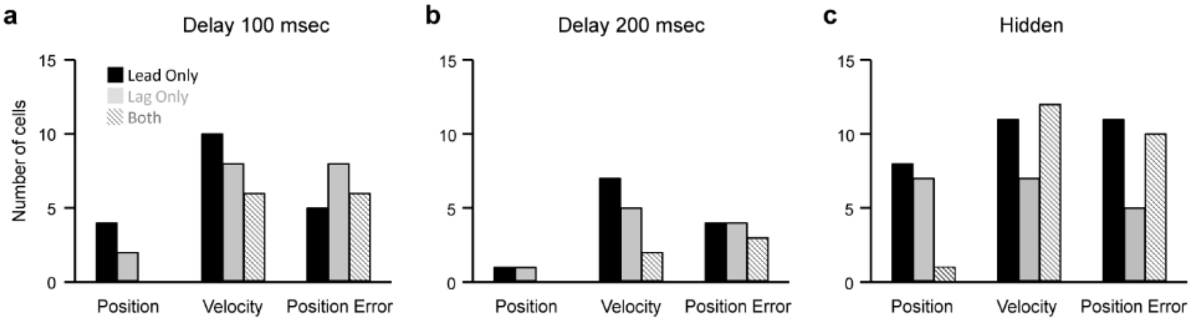

Supplementary Figure 1. Distribution of lead/lag encoding in the SS discharge of Purkinje cells for each behavioral parameter. a and b) Encoding properties for Purkinje cells recorded during both the baseline and either the 100 msec delay or 200 msec delay conditions, respectively. c) Encoding properties for cells recorded the baseline and the hidden condition.

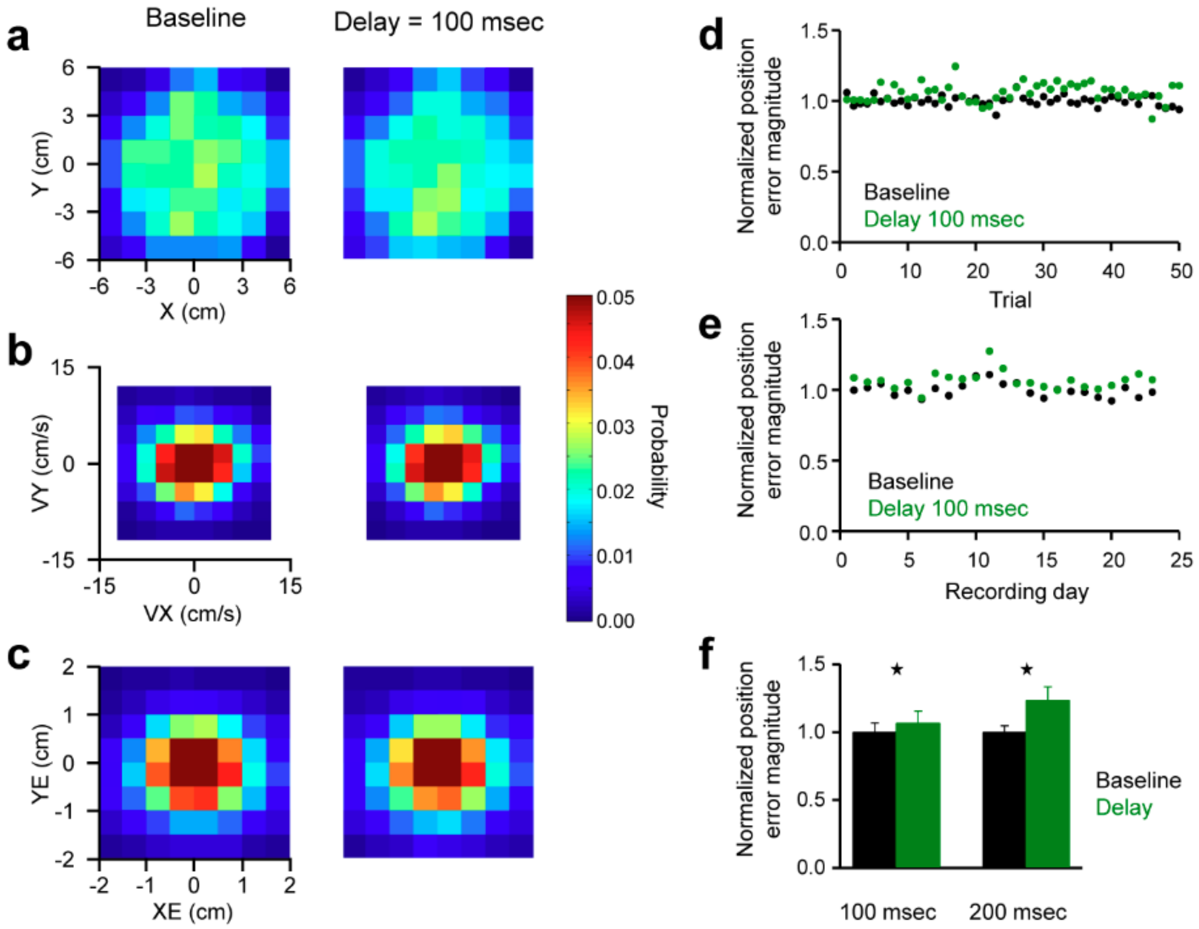

Supplementary Figure 2. Behavior during baseline and delayed cursor conditions. Average probability densities for cursor position (a), velocity (b), and position error (c) across all recording sessions for both baseline (left column of a-c) and the 100 msec delay (right column of a-c) conditions. Average probability values are indicated by color bar. Both baseline and delay conditions result in similar coverage of the parameter workspaces with no significant differences in the probability densities for position ($F(1,127) = 0.67$, $p = 0.42$, ANOVA), velocity ($F(1,127) = 0.08$, $p = 0.77$, ANOVA), and position error ($F(1,127) = 0.95$, $p = 0.33$, ANOVA). $n = 64$ bins for each probability density. Similar results were observed for the 200 msec delay condition (position: $F(1,127) = 0.52$, $p = 0.52$, velocity: $F(1,127) = 0.12$, $p = 0.73$, position error: $F(1,127) = 0.22$, $p = 0.64$, ANOVA, data not shown). d) Average position error magnitude over 50 trials for baseline (black circles) and 100 msec delay (green circles) show no evidence for adaptation (baseline, $\rho = -0.12$ $p = 0.41$, Pearson's correlation, delay = 100, $\rho = 0.07$, $p = 0.63$). For each

monkey, position error magnitude was normalized to the average position error magnitude across trials in the baseline condition. Similar results were observed in the 200 msec delay condition ($\rho = 0.37$, $p = 0.12$, Pearson's correlation, data not shown). e) Average position error magnitude over 23 recording days for baseline (black circles) and 100 msec delay (green circles) shows no evidence for adaptation (baseline, $\rho = -0.27$ $p = 0.22$, Pearson's correlation, delay = 100, $\rho = -0.03$, $p = 0.89$). Similar results were observed in the 200 msec delay condition, with performance error actually tending to increase over trials ($\rho = 0.47$ $p = 0.0006$, Pearson's correlation, data not shown). f) Average normalized position error magnitude in 100 msec (solid bars) and 200 msec delays (dashed bars), illustrating a significant increase in position error in both delay conditions (delay = 100 msec, $t(43) = -4.89$, $p < 0.001$, paired Student t-test, delay = 200 msec, $t(18) = -12.74$, $p < 0.001$, paired Student t-test).

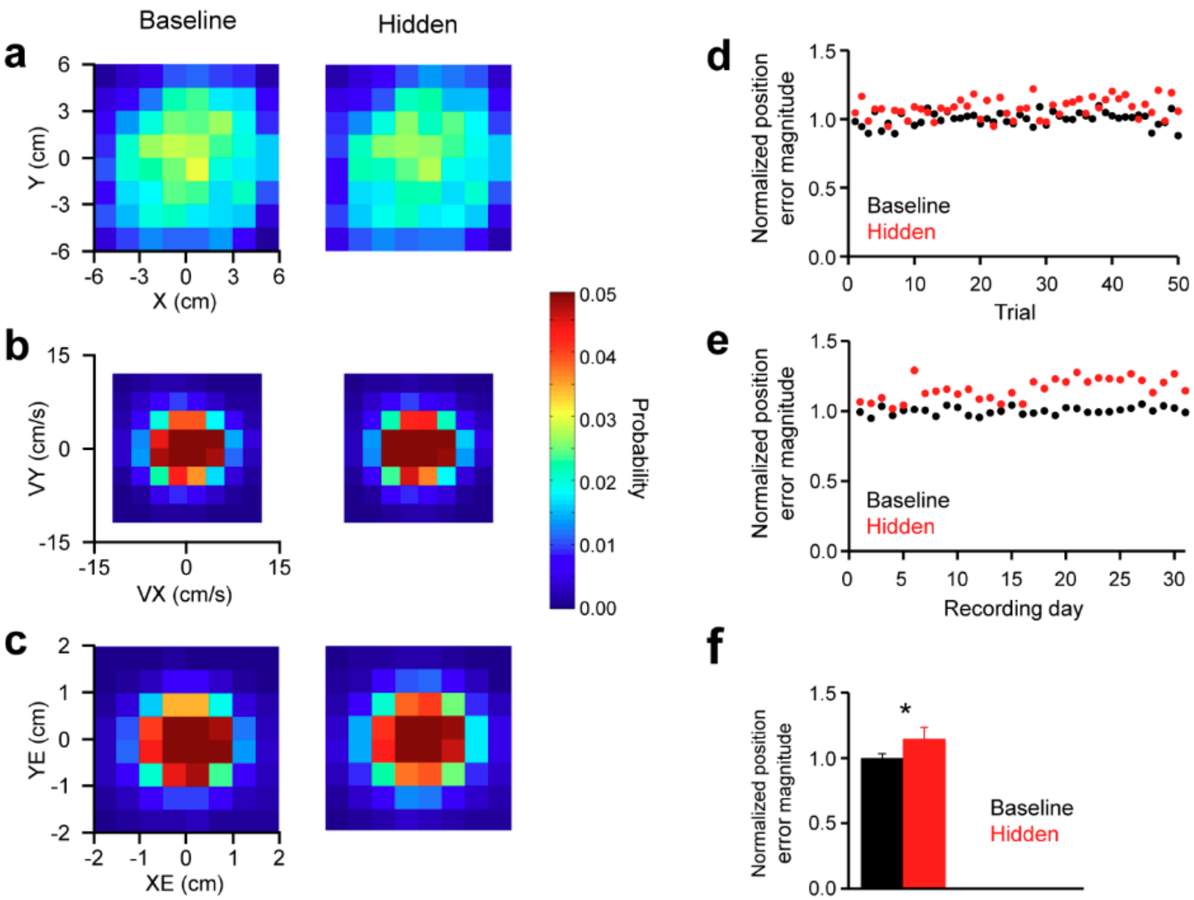

Supplementary Figure 3. Behavior during baseline and hidden cursor conditions. Average probability densities for cursor position (a), velocity (b), and position error (c) across all recording sessions for both baseline (left column of a-c) and the hidden cursor (right column of a-c) conditions. Average probability values are indicated by color bar. There is no significant change in the probability densities for position ($F(1,127) = 1.74$, $p = 0.19$, ANOVA), velocity ($F(1,127) = 0.02$, $p = 0.90$, ANOVA), or position error ($F(1,127) = 0.002$, $p = 0.97$, ANOVA). n = 64 bins for each probability density. d) Normalized position error magnitude over trials for baseline (black circles) and hidden cursor (red circles) illustrates no evidence for improvement of performance across trials, with mean performance error actually tending to increase over trials for the hidden cursor condition (baseline, $\rho = 0.16$ $p = 0.27$, Pearson's correlation, hidden, $\rho = 0.29$, $p = 0.03$). e) Normalized position error magnitude over recording days for baseline (black circles) and hidden cursor (red circles) illustrates no evidence for adaptation, with mean

performance error increasing over recording days for the hidden cursor condition (baseline, $\rho = 0.29$ $p = 0.11$, Pearson's correlation, hidden, $\rho = 0.62$, $p = 0.0002$). f) Normalized position error magnitude illustrates a significant increase in position error in the hidden cursor condition ($t(35) = -9.12$, $p < 0.001$, paired t-test).
